# *Trypanosoma brucei gambiense* Adaptation to Different Mammalian Sera Is Associated with *VSG* Expression Site Plasticity

**DOI:** 10.1371/journal.pone.0085072

**Published:** 2013-12-23

**Authors:** Carlos Cordon-Obras, Jorge Cano, Dolores González-Pacanowska, Agustin Benito, Miguel Navarro, Jean-Mathieu Bart

**Affiliations:** 1 Instituto de Parasitología y Biomedicina "López-Neyra", CSIC, Consejo Superior de Investigaciones Científicas, Granada, Spain; 2 Centro Nacional de Medicina Tropical, Instituto de Salud Carlos III, Madrid, Spain; Federal University of São Paulo, Brazil

## Abstract

*Trypanosoma brucei gambiense* infection is widely considered an anthroponosis, although it has also been found in wild and domestic animals. Thus, fauna could act as reservoir, constraining the elimination of the parasite in hypo-endemic foci. To better understand the possible maintenance of *T*. *b. gambiense* in local fauna and investigate the molecular mechanisms underlying adaptation, we generated adapted cells lines (ACLs) by *in vitro* culture of the parasites in different mammalian sera. Using specific antibodies against the Variant Surface Glycoproteins (VSGs) we found that serum ACLs exhibited different VSG variants when maintained in pig, goat or human sera. Although newly detected VSGs were independent of the sera used, the consistent appearance of different VSGs suggested remodelling of the co-transcribed genes at the telomeric Expression Site (VSG-ES). Thus, Expression Site Associated Genes (*ESAG*s) sequences were analysed to investigate possible polymorphism selection. *ESAGs* 6 and 7 genotypes, encoding the transferrin receptor (TfR), expressed in different ACLs were characterised. In addition, we quantified the *ESAG6/7* mRNA levels and analysed transferrin (Tf) uptake. Interestingly, the best growth occurred in pig and human serum ACLs, which consistently exhibited a predominant *ESAG7* genotype and higher Tf uptake than those obtained in calf and goat sera. We also detected an apparent selection of specific *ESAG3* genotypes in the pig and human serum ACLs, suggesting that other *ESAGs* could be involved in the host adaptation processes. Altogether, these results suggest a model whereby VSG-ES remodelling allows the parasite to express a specific set of *ESAGs* to provide selective advantages in different hosts. Finally, pig serum ACLs display phenotypic adaptation parameters closely related to human serum ACLs but distinct to parasites grown in calf and goat sera. These results suggest a better suitability of swine to maintain *T*. *b. gambiense* infection supporting previous epidemiological results.

## Introduction

Human African Trypanosomiasis (HAT) is caused by two subspecies of the hemoflagellate *Trypanosoma brucei*: *T*. *b. gambiense* and *T*. *b. rhodesiense*, both transmitted by the tsetse fly (Diptera; Glossinidae; *Glossina* sp.), their only known vector. Despite their high biological and genetic similarity, *T*. *b. gambiense* and *T*. *b. rhodesiense* present phenotypic differences observed at clinical and epidemiological levels [1–3]. *T*. *b. rhodesiense* is considered a zoonosis and triggers in humans an acute and severe disease which leads to fatality in weeks or months if untreated. *T*. *b. gambiense* is considered an anthroponosis, causing a chronic wasting disease and is responsible for most of HAT cases [4–6]. Assuming that the *T*. *b. gambiense* reservoir is mainly human, control programs and elimination initiatives have been focused on the early detection of the parasite in this host [7–11]. However, this parasite has also been detected in a wide range of domestic and wild mammalian species and several authors consider these infections relevant for the maintenance of the parasite in the epidemiological cycle under hypo-endemic conditions, where no human cases have been detected for a long time [12–15]. 

To investigate the role played by local fauna as reservoir of *T*. *b. gambiense*, several direct or indirect approaches have been implemented. In the past, some authors attempted to compare, using molecular markers, genotypes circulating between humans and animals harbouring *T*. *b. gambiense*. Despite epidemiological evidence for the parasite in others mammals, the possibility of transmission from animals to human and the implication of these alternative hosts in the epidemiological cycle of the disease are still controversial [16,17]. 

Host ability to allow the parasite survival for long periods is a requirement to define new reservoirs. Thus, *in vivo* follow up of infected animals can be performed to evaluate *T*. *b. gambiense* maintenance in different candidate species [18]. This type of studies has been hampered due to certain associated limitations, such as risk of environmental contamination, low sensitivity of available tests to detect the parasite, parasitaemia fluctuation due to the host immune response, difficult isolation of the parasite to perform further analyses and absence of human controls due to obvious ethical reasons. 

To better control the growth conditions of the parasite and perform cellular and molecular analyses, *in vitro* approaches are preferred. Such approaches have already been applied to study *T*. *b. brucei* adaptation to different mammalian sera [19,20]. These studies focused on Expression Site Associated Genes (*ESAG*s) since they are good candidates for being involved in adaptation because of the large repertoire of families and different alleles. In addition, they are polycistronically transcribed in the telomeric Expression Site (ES) along with the gene that codifies the protein involved in antigenic variation: Variant Surface Glycoprotein (VSG) [21,22]. *T*. *b. brucei* has about 15 VSG-ESs although only one is transcribed at a given time while the others remain repressed, providing the expression of a particular combination of *ESAG*s in a mutually exclusive manner [23]. The transcriptional activation/inactivation of genes in the VSG-ESs is a highly regulated mechanism [24–27], potentially allowing the parasite to quickly respond to any environment change. 

Several studies showed that the genes coding for the transferrin receptor (TfR) were the two *ESAG*s closest to the promoter region, *ESAG6* and *7* [28–30]. In *T*. *b. brucei*, the different copies of *ESAG6/7* sequences are highly polymorphic in regions corresponding to transferrin (Tf) binding sites [31,32]. A controversial hypothesis, still under debate, proposed that the genetic variability of this receptor would provide the parasite with a range of affinities for Tf from different mammalian hosts, which may differ by 30% in amino acid sequence [19,33–35]. Comparable studies have never been conducted for *T*. *b. gambiense* and little is known about *ESAG* sequences and the telomeric *VSG*s that are expressed, since complete VSG-ESs sequences of this parasite are not available [36]. 

Field epidemiological data suggest that a peri-domestic *T*. *b. gambiense* life cycle, involving mammals such as pigs and goats, might occur in West Africa [12,15,37]. We generated serum-adapted cell lines (ACLs) and analysed associated features to investigate the ability of this parasite to grow in different mammalian sera. Initial analysis showed a correlation between adaptation to different sera and expression of new VSGs, suggesting VSG-ES remodelling. Further analyses of *ESAG6*, *ESAG7* and *ESAG3* genotypes, TfR gene expression and Tf uptake were conducted to investigate the molecular mechanisms underlying adaptation responses in *T*. *b. gambiense*. 

## Results and Discussion

### *T*. *b. gambiense* adapts to grow in different mammalian sera

**Figure 1:**
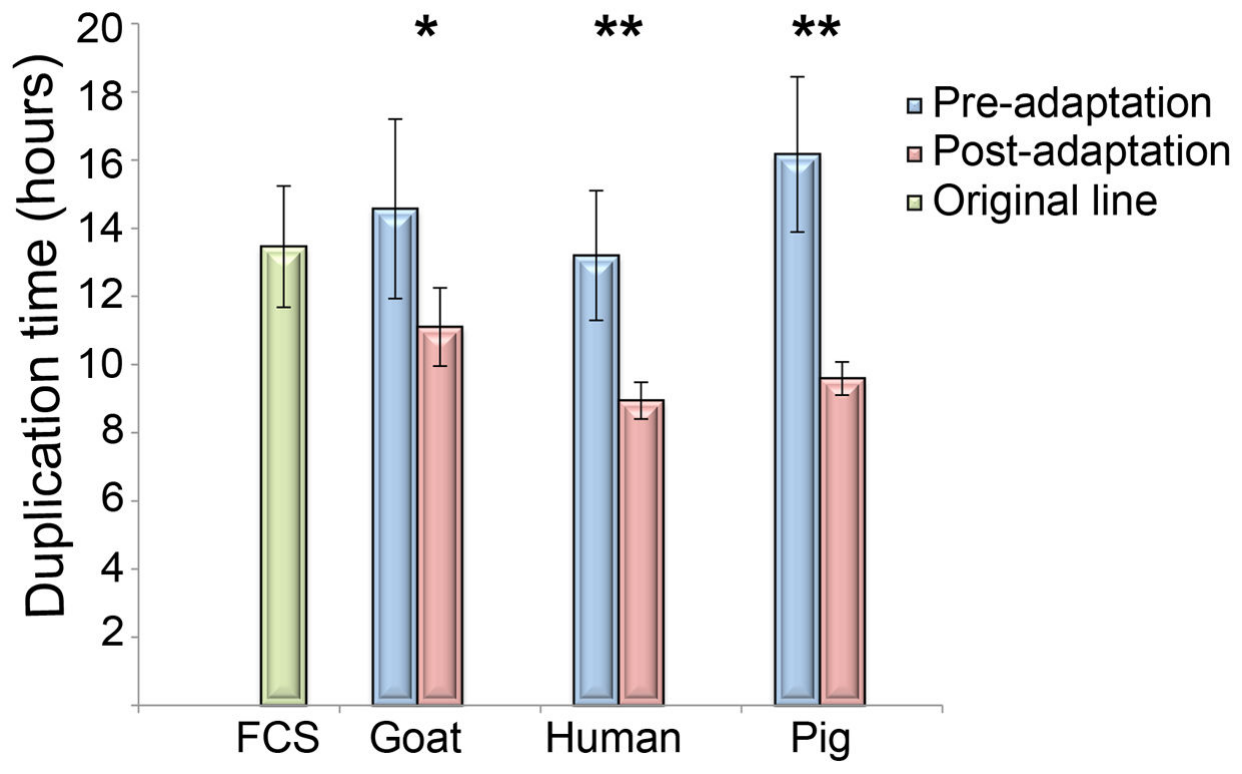
Duplication time of ACLs after adaptation. Histogram showing the duplication time of *T*. *b. gambiense* (ELIANE strain) before and after 25 passages (75-105 days) in HMI9 supplemented with 20% of different mammalian sera. Mean ± SD of at least three independent experiments is shown. Student´s t-test showed significant differences in human and pig serum ACLs between before and after adaptation (* p<0.05; ** p<0.01). FCS: Foetal Calf Serum, ACL: adapted cell line.

Our first evaluation of the adaptation process was to measure the growth rate of cell lines maintained in different sera. Three goat serum (GS1, GS2 and GS3), three pig serum (PS1, PS2 and PS3) and four human serum (HS1, HS2, HS3 and HS4) independent adaptation experiments were performed. Duplication time of the ACLs, after twenty-five passages in the corresponding serum, was compared to the same line before adapting, i.e. at the initial two passages (Figure 1). Human and pig serum ACLs exhibited a significant increase in the rate of proliferation, with a faster duplication time (from 13.2 ± 2.28 to 8.94 ± 0.49 hours in human serum and from 16.17 ± 1.9 to 9.59 ± 0.53 hours in pig serum, p<0.01), suggesting an efficient adaptation. However, goat serum ACLs showed a minor increase in proliferation rate with a smaller reduction in the duplication time (from 14.57 ± 2.63 to 11.1 ± 1.14 hours, p<0.05). Pig serum ACLs exhibited the faster proliferation rate after the adaptation (40.6% lower than pre-adapted line) whereas goat serum ACLs showed a lower reduction in the duplication time (23.8%). The original line, maintained in foetal calf serum (FCS), presented a duplication time of 13.5 ± 1.78 hours.

An effective reservoir should be able to harbour the pathogen for long-term periods while the parasite retains the capacity to infect other hosts. It is widely assumed that *T*. *b. gambiense* has a constitutive resistance to normal human serum (NHS), contrary to *T*. *b. rhodesiense*, which acquires resistance to NHS only when the SRA (Serum Resistance Associated) gene, a particular *ESAG*, is expressed from the active VSG-ES [38–42]. Once adapted, our cell lines were cloned and tested against NHS to address whether, even after a long-term passage in other mammal sera, *T*. *b. gambiense* ACLs was still resistant to human serum. All the clones analysed (27 from calf, 17 from pig and 46 from goat sera) were fully resistant to NHS, while sensitive *T*. *b. brucei* controls systematically were lysed within 16 hours, confirming the constitutive nature of the NHS resistance in *T*. *b. gambiense*. This feature would potentially enable this parasite to infect humans after being maintained in animal cycles.

A previous study on the maintenance of *T*. *b. gambiense* in experimentally infected pigs under controlled conditions showed that *T*. *b. gambiense* causes an asymptomatic infection in this species evolving towards spontaneous cure in 3-4 months [18]. However, we developed an *in vitro* culture system that allows the parasites to adapt to different animal sera, which permitted us to carry out molecular analyses of these ACLs under controlled conditions. In addition, our *in vitro* approach overcomes selection due to the host immune response, physiological changes or individual animal variability, allowing us to analyse molecular aspects associated only with the adaptation process.

### Adaptation to mammalian sera results in selection of new VSG*s*

**Figure 2:**
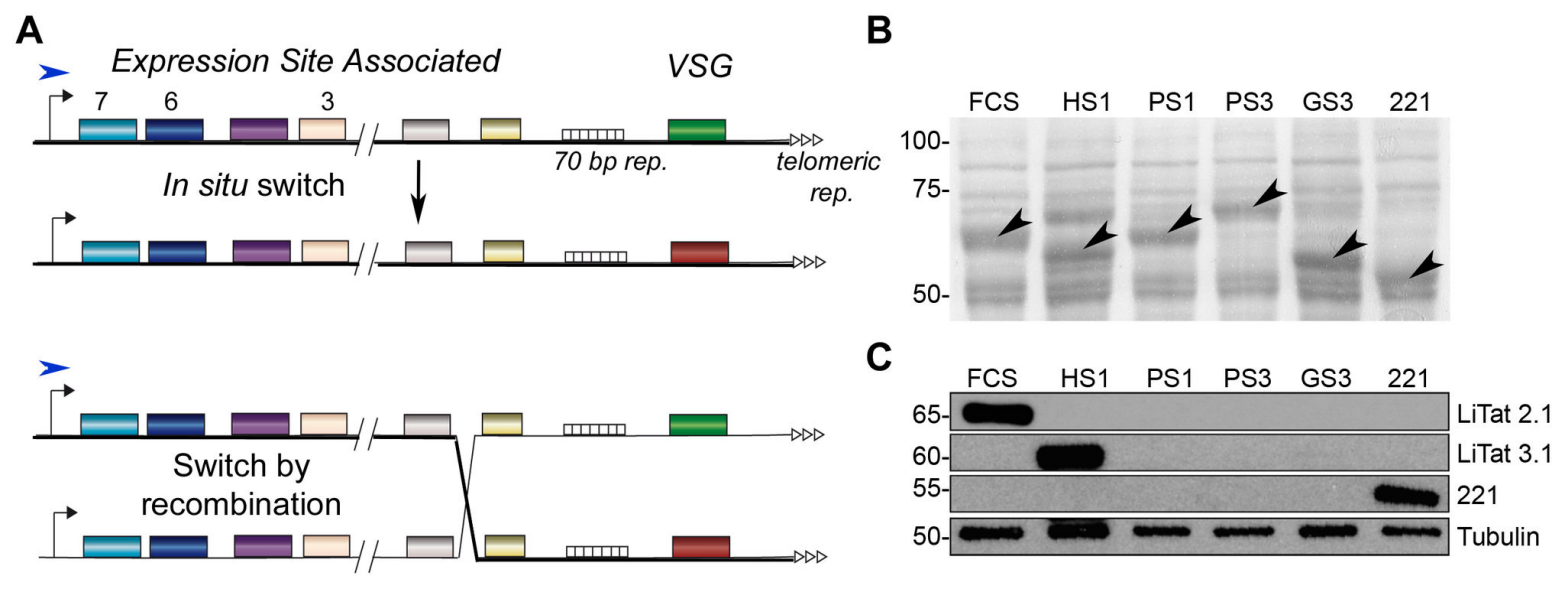
VSG expression in ACLs.﻿ (A) Schematic representation of a canonical *VSG*-Expression Site and the two main mechanisms of antigenic variation in *T. brucei*. Black arrows indicate the promoter sequence and blue arrows the active start transcription site. The thick black line mirrors the transcribed genes. The striped boxes are the 70-base-pair repeats upstream the *VSG* gene and white triangles represent telomeric repeats. Coloured boxes denote *ESAG* and *VSG* genes. Active VSG-ES can be silenced and another telomeric VSG-ES becomes active by *in* *situ* switch (above). Alternatively recombination events in any part (or parts) of the active VSG-ES can occur by homologous recombination, inserting new gene/s from another VSG-ES or non-telomeric locations. (B) Ponceau staining of total protein extract from ACLs showing different VSGs (marked with an arrow). Equivalent amount of 5 x10^6^ parasites was loaded per well. Molecular weight is expressed in kDa. (C) WB analysis of VSGs expressed by different ACLs using anti-LiTat 2.1, anti-LiTat 3.1 and anti-221 antibodies [25]. Anti-Tubulin was used as loading control. Equivalent amount of 1 x 10^5^ parasites was loaded per well. ACL: adapted cell line. FCS: foetal calf serum, GS1/2/3: goat serum (adaptation experiments 1, 2 or 3), HS1/2/3/4: human serum (adaptation experiments 1, 2, 3 or 4), PS1/2/3: pig serum (adaptation experiments 1, 2 or 3), WB: Western Blot.

Previous work in *T*. *b. brucei* suggested that the parasite’s ability to adapt to various mammalian hosts is linked to the differential expression of polymorphic genes of the VSG-ESs, a polycistronically transcribed locus with high genetic plasticity [22,43]. To investigate the role of VSG-ESs in the adaptation of *T*. *b. gambiense* to different hosts, we first analysed the expressed VSG in the different ACLs, as a marker of possible VSG-ES changes. The VSG is a highly abundant protein allowing the direct visualization in total protein extracts of the ACLs. Figure 2B shows an abundant band that varies in size among different lines, suggesting that the VSGs expressed in the ACLs were distinct from those expressed in the original line, maintained in FCS. The high intensity band at 70 kDa observed in the HS1 line was identified by peptide mass fingerprinting as contaminant human albumin.

To characterize these prominent bands, we generated antibodies against the VSGs expressed in the original cell line (LiTat 2.1) and HS1 line (LiTat 3.1) (see Material and Methods). Western Blot (WB) analysis using a mouse monoclonal antibody (mAb) against the original VSG suggested specificity since it exclusively recognizes the VSG present in the FCS ACL (Figure 2C). Likewise, the rabbit antiserum developed against the amino terminal fragment of the LiTat 3.1 only recognized the VSG expressed in the HS1. Although we did not isolate and characterize other expressed VSGs in pig and goat serum ACLs, the different sizes observed by protein staining and the lack of detection by WB using anti-LiTat 2.1 and anti-LiTat 3.1 antibodies suggested that these protein bands correspond to different VSGs. Thus, we detected at least five distinct VSGs during the adaptation assays.

**Figure 3:**
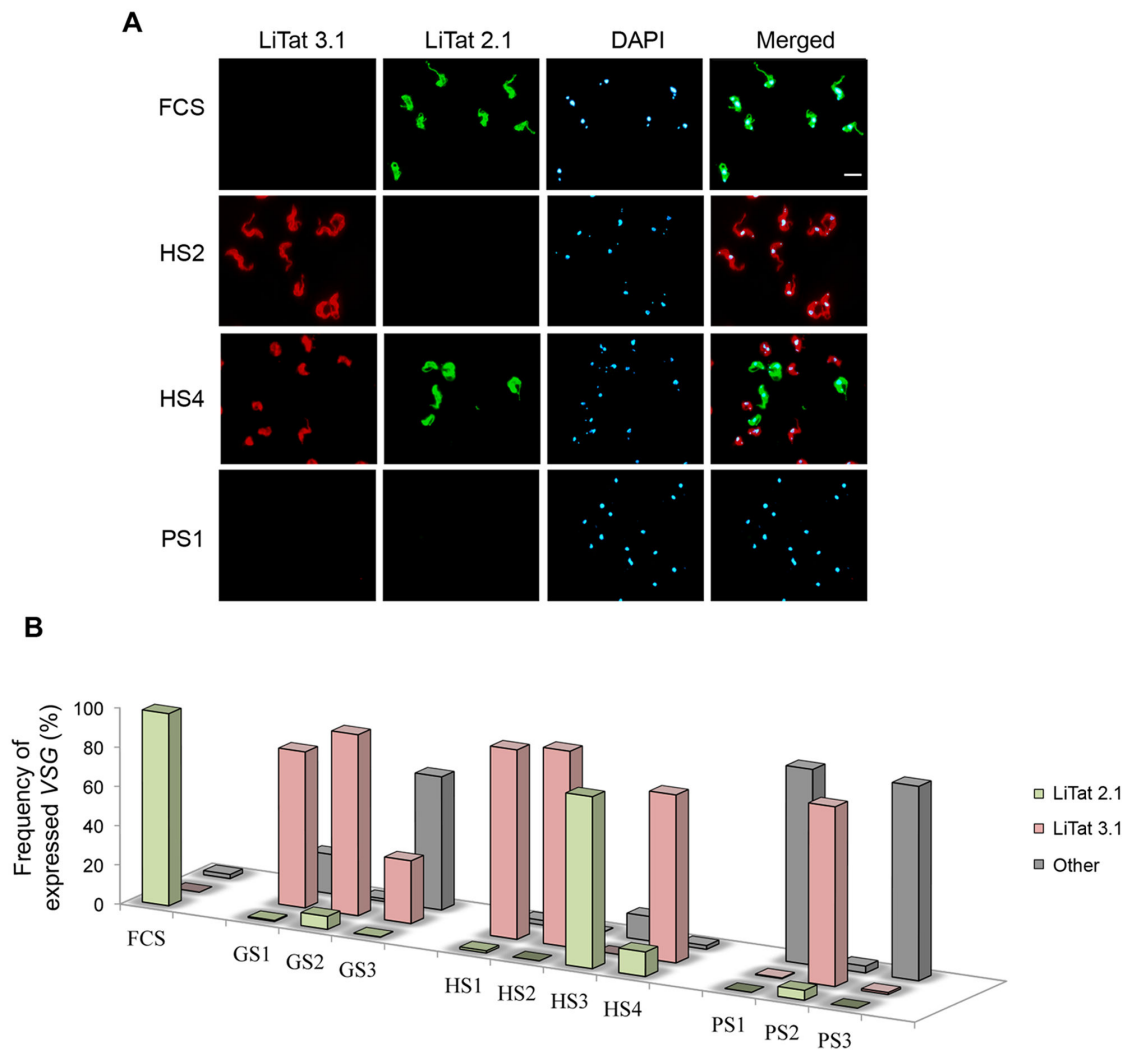
Antigenic variation in ACLs. (A) Immunofluorescence analysis of different *T*. *b. gambiense* ACLs. Monoclonal antibodies anti-LiTat 2.1 VSG and rabbit antiserum anti-LiTat 3.1 VSG were used. White bar represents the scale, set at 5 µm. (B) Frequency of expressed VSG (LiTat 2.1, LiTat 3.1 or other) in ACLs from goat, pig, human and calf sera. Cells were manually counted by IF (n≈500 per sample). FCS: foetal calf serum, GS1/2/3: goat serum (adaptation experiments 1, 2 or 3), HS1/2/3/4: human serum (adaptation experiments 1, 2, 3 or 4), PS1/2/3: pig serum (adaptation experiments 1, 2 or 3).

Immunofluorescence (IF) experiments were also performed with the specific mAb anti-LiTat 2.1 (Figure 3). This analysis revealed that nine out of ten ACLs expressed a different dominant VSG. Parasites selected in pig and goat sera yielded a population where at least 93.5% of cells expressed a VSG distinct from the original one. Three out of the four human serum ACLs (lines HS1, HS2 and HS4), also expressed different VSGs (100%, 96.5% and 87.4% of VSG change respectively), and only a single human serum ACL (HS3) maintained the original LiTat 2.1. 

IF analysis using anti-LiTat 3.1 showed that six out of the ten new ACLs expressed on their surface LiTat 3.1 as the more frequently VSG: three of human ACLs (99.8%, 93% and 85.6%), two of goat (79.5% and 92.2%) and one of pig (91.5%). The two remaining pig ACLs expressed neither LiTat 2.1 nor LiTat 3.1, while only 30% of the GS3 expressed LiTat 3.1 (Figure 3B). 

A non-clonal population was used for these studies since it offers a more suitable initial diversity for selection, as reported previously in *T*. *b. gambiense* [44]. In spite of this, IF analysis showed the stable expression of the LiTat 2.1 VSG in the original line after culture with FCS for several months. LiTat 3.1 was the most common VSG variant detected, probably due to its presence in the original population and further VSG-ES selection. 

These results suggest that the type of VSG expressed is independent of the serum used for selection. However, in all cases but one (HS3), we detected a switch in the VSG expressed in the ACLs suggesting that VSG-ES changes in the co-expressed *ESAG*s might be involved in the adaptation process.

### *ESAG7* shows a specific genotype in pig and human ACLs

Differential VSG expression after growth selection using different animal sera has been previously reported in *T*. *b. brucei* [33,34]. In particular, expression of polymorphic *ESAG*s has been proposed to explain the adaptation mechanism to mammalian sera. Although this hypothesis is still not definitively proved, expression of distinct *ESAG*s 6 and 7, encoding the polymorphic TfR [30], may have significant effects on the binding affinity for diverse mammalian Tfs [19,34]. 

**Figure 4:**
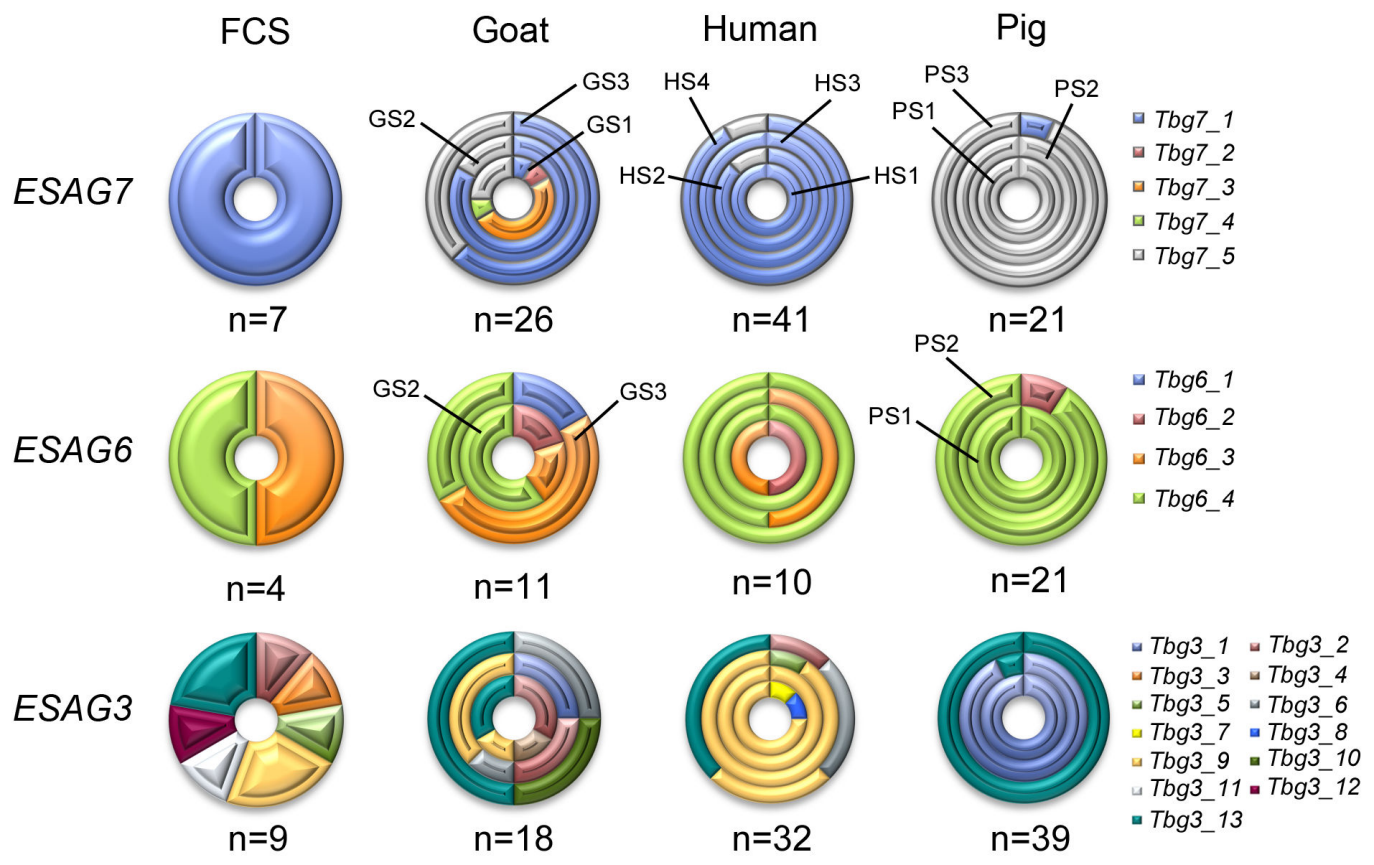
*ESAG* diversity in ACLs. Graphic representation of *ESAG* genotype diversity. Each ring is divided in 2-4 inner circles representing each independent experiment. Unless otherwise is indicated, ACLs distribution in each ring is as indicated in *ESAG7* row. Each genotype is represented in a different colour according to indicated in the graph. Partial ORF was used for DNA genotyping (673-676 bp in *ESAG6/7* and 853 bp in *ESAG3*). FCS: foetal calf serum, GS1/2/3: goat serum (adaptation experiments 1, 2 or 3), HS1/2/3/4: human serum (adaptation experiments 1, 2, 3 or 4), PS1/2/3: pig serum (adaptation experiments 1, 2 or 3).

In order to investigate whether adaptation in the ACLs generated in this study could be associated with specific TfR selection, we first characterized the cDNA sequence diversity of *ESAGs* 6 and 7 in all our ACLs (Figure 4 and Table 1). We utilized a single set of primers to amplify both genes and after sequencing the PCR fragments we were able to distinguish each gene and their genotypes. 

**Table 1:** ESAG6/7 genotype diversity.

**Genotype**	**FCS**	**GS1**	**GS2**	**GS3**	**HS1**	**HS2**	**HS3**	**HS4**	**PS1**	**PS2**	**PS3**	**Total**
*Tbg7_1*	7	1	5	5	10*	7*	11*	11*			1	**58**
*Tbg7_2*		1										**1**
*Tbg7_3*		6*										**6**
*Tbg7_4*		1										**1**
*Tbg7_5*		3	1	3		1		1	2*	4*	14*	**29**
*Tbg6_1*				1								**1**
*Tbg6_2*			1		1					1		**3**
*Tbg6_3*	2		1	3	1		2					**9**
*Tbg6_4*	2		3	2		3	2	1	10	10		**33**
**Protein TfR**												
*Tbg7_1/2*	7	2	5	5	10	7	11	11			1	**59**
*Tbg7_3*		6										**6**
*Tbg7_4*		1										**1**
*Tbg7_5*		3	1	3		1		1	2	4	14	**29**
*Tbg6_1*				1								**1**
*Tbg6_2/3/4*	4		5	5	2	3	4	1	10	11		**45**
**Total**	**11**	**12**	**11**	**14**	**12**	**11**	**15**	**13**	**12**	**15**	**15**	**141**

Summary of *ESAG6/7* genotypes distribution amongst ACLs. Data represent the number of times a given genotype was obtained during genotyping analysis. Significant association between serum and genotype, assessed by Likelihood Ratio Test completed with residues analysis, is marked with an asterisk (*). For analysis, lines were grouped by serum species. Partial ORF was used for DNA genotyping (673-676 bp). Only TfR region was used in alignments at protein level. FCS: foetal calf serum, GS1/2/3: goat serum (adaptation experiments 1, 2 or 3), HS1/2/3/4: human serum (adaptation experiments 1, 2, 3 or 4), PS1/2/3: pig serum (adaptation experiments 1, 2 or 3).

**Figure 5:**
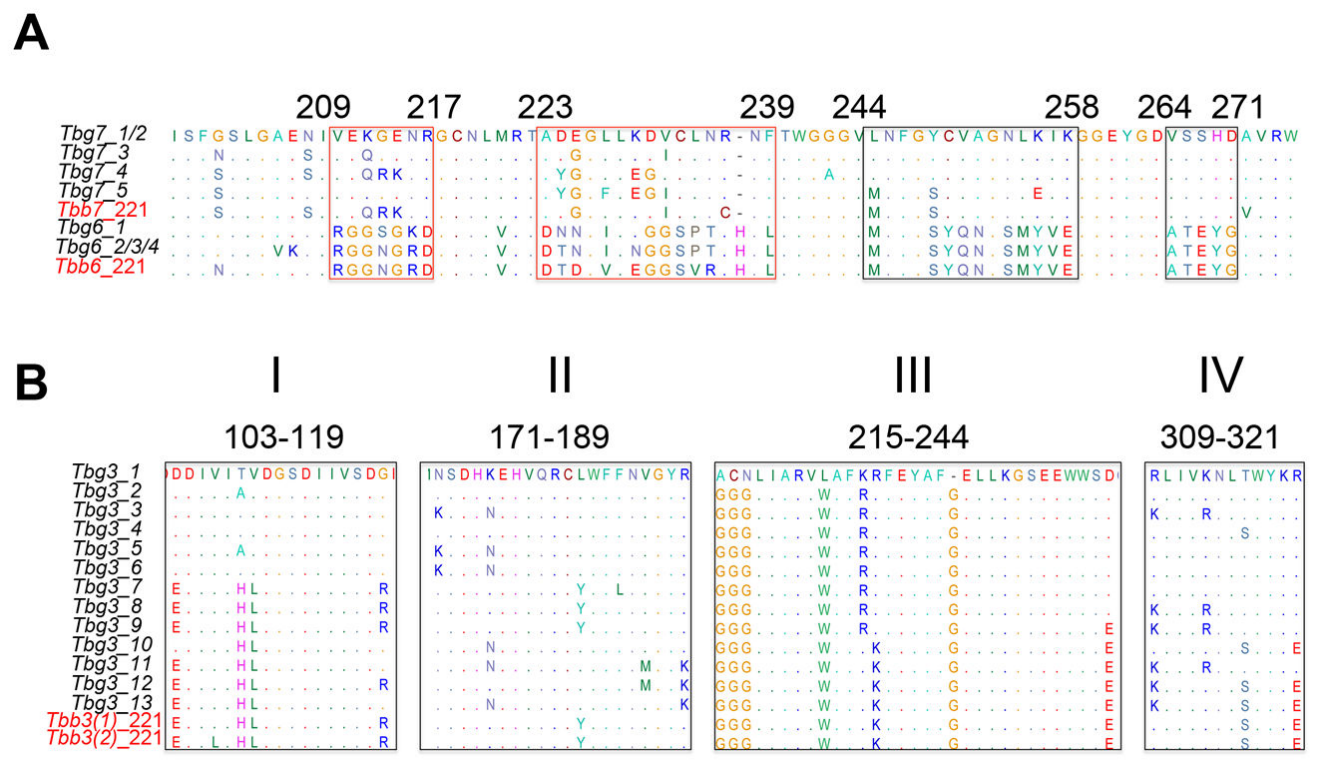
Alignment of Tf binding sites (ESAG6/7) and polymorphic ESAG3 regions. Amino acid alignment of polymorphic regions of ESAG6/7 (A) and ESAG3 (B). Tf binding sites are highlighted with red squares. Other polymorphic regions of ESAG6/7 are marked with black boxes. Polymorphic regions of ESAG3 are shown. I: S103-E119; II: L171-K189; III: A/G215-D/E244; IV: A309-R/E321. *T*. *b. brucei* ESAGs 6, 7 and 3 are shown in red.

Overall, we obtained five *ESAG7* and four *ESAG6* genotypes, defined as single nucleotide changes in the sequence. Table 1 and Figure 4 show the genotype frequency obtained for each ACL and Figure S1A shows a clustering tree for both genes. Average DNA diversity was 4.56% (ranging from 1.65% to 6.07%) and 3.88% (from 0.15% to 7.58%) for *ESAG7* and *ESAG6* respectively (in terms of number of nucleotide substitutions per polymorphic site). Interestingly, protein alignments of the Tf binding regions yielded only two types of *ESAG6* out of four genotypes (one represented only by a single clone), while four *ESAG7* proteins corresponding to five different genotypes were detected (Figure 5A and Table 1).

Low diversity of *T*. *b. gambiense ESAG6* has been reported previously [36]. The main genotypes found in our study, *Tbg6_4* and *Tbg6_3*, correspond to type 10 and 7 respectively, described by Young et al. [36]. *Tbg6_1* shares 99% homology (675/676 nucleotides) with type 8 and *Tbg6_2* is highly similar to types 7 and 5 (99% homology, 674/676 in both cases). The authors suggested that *T*. *b. gambiense* shows less *ESAG6* diversity because is highly adapted to the human host. Our data also showed less variability in *ESAG6* genotypes suggesting that most *T*. *b. gambiense* VSG-ESs contain *ESAG6* copies encoding high-affinity TfR for human Tf. 

Contrary to *ESAG6*, we detected an interesting correlation between the serum used for selection and the *ESAG7* type. Genotype *Tbg7_5* correlated to pig serum, whereas *Tbg7_1* was significantly associated with human serum (p< 0.01). Conversely, adaptation in goat serum yielded a more diverse pattern (all four *ESAG6* and five *ESAG7* genotypes were found in goat serum ACLs). Van Luenen et al. [34] investigated in detail changes in *T*. *b. brucei ESAG6/7* expression upon adaptation to growth in dog serum. Their data showed that the parasite might respond to changes in serum either by *in situ* switching to another VSG-ES to express a new *ESAG6/7* with higher affinity for dog Tf, or by upregulation of *ESAG7* expression from an inactive VSG-ES promoter-proximal region, resulting in expression of different *ESAG7* genotypes. A similar upregulation of *ESAG6/7* from an inactive VSG-ES was described after deletion of these genes from the active VGS-ES [45]. We also detected a major *ESAG7* mRNA type in some ACLs and others less abundant, suggesting a residual expression from inactive VSG-ESs. In order to investigate whether the different *ESAG7* mRNA sequences from ACLs were due to low expression of inactive VGS-ESs or to population variability [46], we cloned and sequenced the expressed *ESAG6/7* of two clones obtained from the human serum ACL HS2 and two from HS3. While most of the clones expressed a single *ESAG6/7* genotype, in the clone 2 from HS3, we found two different *ESAG7s* genotypes, one more abundant corresponding to that detected in the HS3 (data not shown). These data suggest that polymorphisms in *ESAG7* should not be entirely attributed to mixed population expressing different VSG-ESs, but rather to the expression from inactive VSG-ESs.

It has been reported that after *T*. *b. brucei* adaptation to canine serum, *ESAG7* genotypes distinct to the original wild type line were frequently obtained, whereas the *ESAG6* type usually remained constant [34]. These data are consistent with our results, suggesting that *ESAG7* diversity, rather than *ESAG6*, is relevant for the adaptation to different Tfs. 

In summary, specific genotypes of *ESAG7* were identified in human and pig serum ACLs. The selection of these genotypes may confer phenotypic advantages during the adaptation process and be directly related to the increased competency in growth compared to goat and calf serum ACLs (Figure 1).

### Pig ACLs show higher human transferrin uptake

**Figure 6:**
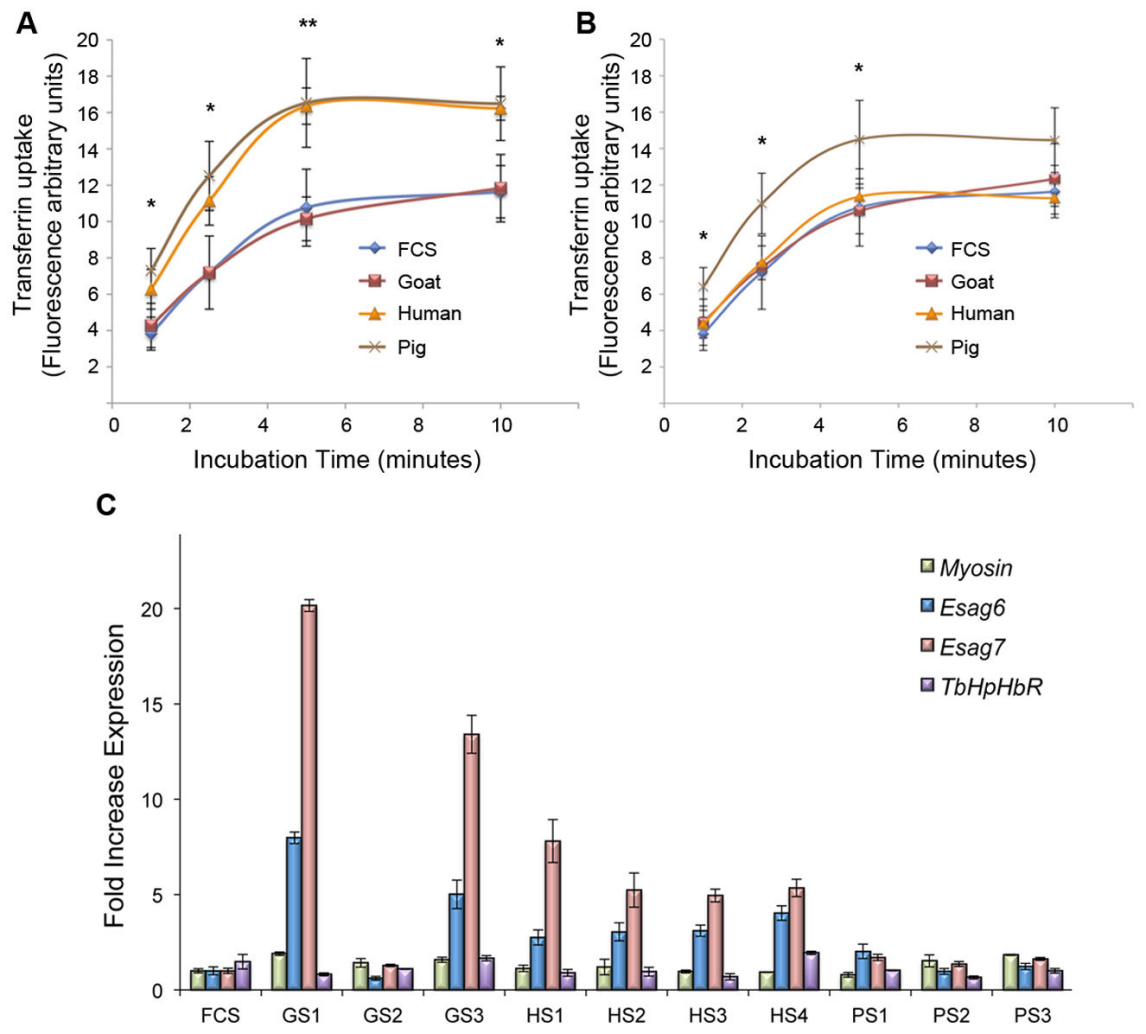
Tf uptake and mRNA *ESAG6/7* expression levels. (A) Tf uptake of different ACLs. Mean ± SD of three (goat and pig) or four (human) sera independent adaptation experiments and three independent measures of calf serum ACL are shown. Uptake is expressed in FAU (fluorescence arbitrary units). One-way ANOVA test was performed (* p< 0.05; ** p<0.01). (B) Normalized Tf uptake of different ACLs. Data from Figure 6A were normalized with dextran uptake at 10 minutes of incubation (data not shown). One-way ANOVA test was performed (* p< 0.05; ** p<0.01). Human holo-Tf (iron saturated) was used at final concentration of 20 μg/ml. (C) Histogram showing relative expression of mRNA measured by qRT-PCR of *ESAG6*, *ESAG7* and *TbHpHbR* in all ACLs. Mean ± SD of two different measures is shown. Expression values are plotted in relative expression units, calculated relative to *ATM* (*PI3Kinase-*like -Tbb927.2.2260-) expression. *Myosin* housekeeping gene is also shown [25]. ACL: adapted cell line, *TbHpHbR*: Haptoglobin-Hemoglobin receptor, FCS: foetal calf serum, GS1/2/3: goat serum (adaptation experiments 1, 2 or 3), HS1/2/3/4: human serum (adaptation experiments 1, 2, 3 or 4), PS1/2/3: pig serum (adaptation experiments 1, 2 or 3).

To gain insight on the role of differential *ESAG6/7* expression in the adaptation process, we analysed Tf uptake in all our ACLs. We performed the experiments with human holo-Tf (iron saturated) since humans are considered the main *T*. *b. gambiense* host. Sub-physiological concentration of Tf was used in order to better track the uptake variations (20 μg/ml, i.e. around 50 fold less holo-Tf relative to normal serum and 10 fold less relative to our culture conditions). Maximum uptake was reached between 5 to10 minutes of incubation in all ACLs (Figure 6A). Human and pig serum ACLs showed the most efficient uptake with 16.25 ± 1.00 and 16.52 ± 2.45 FAU (fluorescence arbitrary units) at 5 minutes of incubation, respectively. Significant differences were detected in Tf uptake when all ACLs were compared. Pair wise *post-hoc* analyses showed that goat serum ACLs did not significantly differ from the original line at any incubation time, whereas pig and human serum ACLs uptake increased by 60% respective to the other lines (p= 0.017 and p= 0.013 at 5 minutes of incubation time, respectively). However, the Tf uptake differences could be due not only to receptor mediated endocytosis variation but also to changes in the endocytic rate. Thus, we carried out fluid-phase endocytosis assays using Alexa Fluor^®^ 488-labelled dextran 10,000 (Molecular Probes) [47] to normalize the transferrin uptake (Figure 6B). After normalization, pig serum ACLs conserved a higher uptake of Tf compared to goat and calf serum ACLs, indicating that at least 36% of uptake increase was not due to differences in the endocytosis rate. Thus, higher Tf uptake in pig serum ACLs could be attributed, at least in part, to the specific *ESAG7* genotype (*Tbg7_5*) expressed in these lines. Normalized Tf uptake values of human serum ACLs, contrary to pig serum ACLs, suggest that a higher endocytic rate is influencing the Tf uptake data. This can explain why calf and human serum ACLs have different Tf uptake while sharing similar *ESAG6/7* genotypes. In addition, increased *ESAG6/7* mRNA levels were consistently detected in human serum ACLs (ranged between 4.95 ± 0.34 to 7.81 ± 1.13 fold relative to original line, Figure 6C), suggesting higher protein levels in the flagellar pocket. Although we have not properly tested this hypothesis by protein detection, the modulated expression of TfR at both mRNA and protein levels have been well described during adaptation, showing that *ESAG6/7* transcript levels largely mirror the protein TfR amount in the flagellar pocket [20,34]. We do not have a satisfactory explanation of why the endocytic rate increases upon culture in presence of human serum, although it is possible that several unknown factors present in human blood, the *T*. *b. gambiense* main reservoir, could provide benefits to the parasite in essential processes such as the endocytic pathway.

GS1 and GS3 showed even higher expression of *ESAG7* mRNA levels than human serum ACLs (20.16 ± 0.31 and 13.4 ± 0.99 fold relative to FCS ACL), although this did not entail a higher Tf uptake. As we did not observe a more efficient adaptation phenotype, we could not attribute this finding to a clone selection as may occur in human serum ACLs. Rather, we hypothesize that continuous culture with goat serum resulted in upregulation of mRNA levels of *ESAG6/7* as previously described in *T*. *b. brucei* using dog serum [20], leading to a diverse expression pattern (Figure 4). 

Although efficient uptake of Tf by the TfR is essential for parasite growth [48], some authors would not consider it as a selection factor under *in vivo* conditions, since even a low affinity receptor could provide the parasites with the low iron requirement they need [49]. Chronic *T*. *b. gambiense* infections last months or even years and the immune response of the host produces a wide range of antibodies against parasite antigens such as TfR [50]. It is known that these anti-TfR antibodies can compete with Tf for binding to TfR [19,51] and Tf uptake inhibition by these means seems especially substantial when a low affinity TfR is expressed [19]. Additionally, indirect evidences suggest that Tf availability in the flagellar pocket, where TfR is expressed, could be up to 30 fold lower compared to its concentration in surrounding medium [20]. Altogether, these data suggest that a high affinity TfR could be important for the parasite to ensure sufficient Tf uptake *in vivo*. As proposed by van Luenen et al. [34] this issue will need further analysis with trypanosomes isolated from wild fauna to elucidate whether there is a differential TfR selection upon parasite adaptation in different mammals. This would be especially difficult in *T*. *b. gambiense*, given the low prevalence reported in the local fauna [12]. 

Recently, the mechanism of *T*. *b. gambiense* resistance to human serum has been discovered [52]. This is consequence of multiple factors as the expression of *TgsGP* gene and the reduction/inactivation of another receptor located in the flagellar pocket: the haptoglobin-hemoglobin receptor (TbHpHbR), involved in trypanolytic factor 1 (TLF-1) internalization [52]. Since no previous molecular studies on *T*. *b. gambiense* adaptation in different mammals are available, we decided to study these factors in our serum ACLs. 

*TgsGP* gene is traditionally used as marker of *T*. *b. gambiense* group 1 [53,54]. This gene was detected by PCR from genomic DNA before and after the adaptation process in all our ACLs (data not shown). On the other hand, we measured the transcripts level of TbHpHbR, whose expression is known to be downregulated in *T*. *b. gambiense* [55]. As this receptor also binds haptoglobin-hemoglobin complexes of the host in order to incorporate heme [56], one could expect that upon non-human mammalian serum adaptation (i.e. in absence of human TLF-1), TbHpHbR levels could vary. Our data, however, demonstrate that the low TbHpHbR mRNA levels represent a constitutive feature of *T*. *b. gambiense* and, contrary to *ESAG6/7* transcripts, remain stable upon adaptation (Figure 6C). This suggests that regardless of the serum used, the amount of heme does not constitute a limiting factor for parasite growth. 

### *ESAG3* specific genotypes are selected in pig and human ACLs

Concomitant to changes in the expression of different *ESAG7* genotypes, the expression of other *ESAGs* may also change, since they are linked to the VSG-ES locus. Thus, we decided to investigate possible variations in the expression of *ESAG3* in the ACLs, located further downstream of the VSG-ES promoter. Although *ESAG3* function is unknown, the predicted protein contains a signal peptide in the N-terminal that putatively targets the protein to the cellular surface, suggesting an interaction with the host environment [22]. In addition, *ESAG3* is present in almost all VSG-ESs of *T*. *b. brucei* [57]. 

Sequence analysis identified thirteen different genotypes (see Figures 4, 5B, S1B and Table 2). Average DNA diversity was 3.7% (ranging from 0.12% to 6.24%), in four polymorphic regions (Figure 5B). Similar to *ESAG6/7*, we detected a more abundant *ESAG3* type in human and pig serum ACLs, suggesting mono-allelic expression from a single active VSG-ES. We observed significant correlation (p< 0.01) between *Tbg3_13* and *Tbg3_1* genotypes in the pig serum ACLs and *Tbg3_9* in human serum ACLs (Table 2). However, HS4 had no predominant genotype and expressed at least four different *ESAG3* types. Calf and goat serum ACLs also showed a diverse expression pattern as occurred in *ESAG6/7* genes (Table S1). The lack of correlation between the expressed VSG and the serum ACLs hinders the interpretation of the *ESAG3* selection in human and pig serum ACLs. Although some *ESAG3* non-telomeric copies can exist as pseudogenes in subtelomeric arrays both in *T*. *b. gambiense* [58] and *T*. *b. brucei* [59], the transcription from VSG-ESs by RNA polymerase I is much more efficient than a possible transcription of genes mediated by RNA polymerase II from chromosomal internal positions [22,60]. Therefore, genotypes from active VSG-ESs are likely the most abundant type in the cDNA utilized as template in our genotyping analysis. For example, *Tbg3_9*, the most common type found in our study, corresponds to an *ESAG3* copy from an expression site (TbgBES11) previously obtained during *T*. *b. gambiense* genome sequencing (GenBank AEX08459.1) [58]. *Tbg3_1*, another major genotype, and *Tbg3_13*, expressed mainly by PS3, share 98% and 97% similarity, respectively, with *ESAG3* from TbgBES17 (GenBank AEX08464.1). On the other hand, residual expression of *ESAG3* from inactive expression sites is unlikely, since the gene is located far away from the promoter. Thus, the expression pattern of *ESAG3* in human and pig serum ACLs together with the similarity with previously reported data suggest that sequences obtained in these ACLs derived from the active VSG-ES. 

**Table 2:** ESAG3 genotype diversity.

**Genotype**	**FCS**	**GS1**	**GS2**	**GS3**	**HS1**	**HS2**	**HS3**	**HS4**	**PS1**	**PS2**	**PS3**	**Total**
*Tbg3_1*			2						11*	13*		**26**
*Tbg3_2*	1	2*	2*					1				**6**
*Tbg3_3*	1											**1**
*Tbg3_4*		1										**1**
*Tbg3_5*	1						1					**2**
*Tbg3_6*			1	1				2				**4**
*Tbg3_7*					1							**1**
*Tbg3_8*					1							**1**
*Tbg3_9*	2	1	3		6*	5*	10*	2*				**29**
*Tbg3 _10*				1								**1**
*Tbg3_11*	1											**1**
*Tbg3_12*	1											**1**
*Tbg3_13*	2	2		2				3		1*	15*	**25**
**Protein**												
*Tbg3_1*			2						11	13		**26**
*Tbg3_2*	1	2	2					1				**6**
*Tbg3_3*	1											**1**
*Tbg3_4*		1										**1**
*Tbg3_5*	1						1					**2**
*Tbg3_6*			1	1				2				**4**
*Tbg3_7*					1							**1**
*Tbg3_8*					1							**1**
*Tbg3_9*	2	1	3		6	5	10	2				**29**
*Tbg3_10*				1								**1**
*Tbg3_11*	1											**1**
*Tbg3_12*	1											**1**
*Tbg3_13*	2	2		2				3		1	15	**25**
**Total**	**9**	**6**	**8**	**4**	**8**	**5**	**11**	**8**	**11**	**14**	**15**	**99**

Summary of *ESAG*3 genotypes distribution amongst ACLs. Data represent the number of times a given genotype was obtained during genotyping analysis. Significant association between serum and genotype, assessed by Likelihood Ratio Test completed with residues analysis, is marked with an asterisk (*). For analysis, lines were grouped by serum species. Partial ORF was used for DNA genotyping (853 bp). FCS: foetal calf serum, GS1/2/3: goat serum (adaptation experiments 1, 2 or 3), HS1/2/3/4: human serum (adaptation experiments 1, 2, 3 or 4), PS1/2/3: pig serum (adaptation experiments 1, 2 or 3).

The apparent *ESAG3* genotypic selection in human and pig serum ACLs, together with the lack of *VSG*-serum correlation could be explained by VSG-ES remodelling. In human and pig serum ACLs, the selected genotypes (*Tbg3_1*, *Tbg3_9* and *Tbg_13*) might be a result of insertions of copies from either non-telomeric sites or other VSG-ESs by gene conversion driven by the high sequence homology (Figure S2) [57,61]. Recombination between VSG-ESs during a selection procedure has been described in *T*. *b. brucei* during *in vitro* selection using dog serum [34] and after adaptation to human HDL [62]. It is possible that some active VSG-ESs observed in this study, including that expressed in the original line containing LiTat 2.1 *VSG*, lack full-length copies of *ESAG3*, allowing the detection of genotypes from non-telomeric sites and leading the diverse patterns found in other ACLs such as calf and goat serum ACLs and HS4. Likewise, in *T*. *b. brucei* five telomeric VSG-ESs with no functional copy of *ESAG3* have been described [57], including BES13/TAR56, which contains a promoter region highly similar to that of the *T*. *b. gambiense* VSG-ESs [36]. 

The predicted protein encoded by genotype *Tbg3_1* associated with PS1 and PS2, was a truncated version due to the presence of a stop codon insertion at the amino acid position 232. This is not consistent with a selection process of this gene and the selection of some particular *ESAG3* genotypes could be a side effect of the selection of other *ESAG*s present in the active VSG-ES. Alternatively, as PS1 and PS2 shared *ESAG6*, 7 and 3 genotypes, but different VSGs, it is possible than the same active VSG-ES is being transcribed in both ACLs (Table S1 and Figure S2). In this case, a recombination event could have occurred downstream the *ESAG3*. The lack of apparent selection in some ACLs and the truncated product encoded by PS1 and PS2 difficult the interpretation of the role of ESAG3 in adaptation. However, we cannot rule out that it may confer an advantage in the adaptation. ESAG3 is a 368 amino acid protein, with an amino terminal signal peptide, which lacks obvious transmembrane or anchoring domains. It has been hypothesized that ESAG3 is released into surrounding medium under undetermined conditions [22]. Secreted proteins have been described as important to parasite-host crosstalk in other organisms such as *Plasmodium berghei, Fusarium oxysporum, Sclerotinia sclerotiorum, Toxoplasma gondii or Bacillus anthracis* [63–66].

In summary, *ESAG3* genotyping suggests two ACLs clusters. The first group (calf and goat serum ACLs) displayed high level of polymorphism, while the second group (pig and human serum ACLs) showed more homogenous genotypes. The former ones probably mirror the expression of non-telomeric copies due to the lack of a functional *ESAG*3 gene in the active VSG-ES. The latter ones appear to select a particular genotype, suggesting expression from the active VSG-ES. 

## Conclusions

*T*. *b. gambiense* was able to efficiently proliferate *in vitro* in all mammalian sera tested. However, appearance of different VSG variants has been consistently observed in this parasite after adaptation, independently of the serum used. This suggests that while changes in the VSG type are not associated to sera selection, expression remodelling of the VSG-ES *ESAG*s is probably required. *ESAG* polymorphism analysis showed a lower degree of variations in pig and human sera than in goat and calf serum ACLs, which is consistent with the more efficient growth in pig and human sera, suggesting swine as a more suitable reservoir than any other animal. 

After adaptation to pig and human sera, these ACLs exhibited an increase in human Tf uptake. In the case of pig serum ACLs, high Tf uptake is associated with the expression of a specific ESAG7 genotype while human serum ACLs showed increased endocytic rate and probably TfR amount based on mRNA levels. However, goat serum ACLs showed a slower growth rate after adaptation, a larger *ESAG6/7* genotype diversity and increased levels of *ESAG6/7* transcripts, but still lower Tf uptake than human and pig serum ACLs. These data suggest a quick modulated adaptation response, as occurs in *T*. *b. brucei*, immediately after being exposed to dog serum [34]. Parasites seem unable to attain a complete and stable adaptation in goat serum, since they still show the short-term quick responses after at least seventy-five days of culture. 

*ESAG3* genotypes also seemed to correlate with the serum utilized in the adaptation, showing specific types expressed mainly in human and pig serum ACLs, whereas in goat serum ACLs a diverse pattern was noticed. As we discussed above, although this selection is probably a side effect of the rearrangement of VSG-ES to select other *ESAG*/s, these data still suggest that the pressure upon sera selection is not only for a specific genotype of *ESAG7* but rather for a combination of *ESAGs*. Supporting this model, a recent article describes a large family of transmembrane receptor-like adenylate cyclases, highly represented by the polymorphic *ESAG*/*GRESAG4*, as a parasite tool to control the immune response of the host [67]. Although, our *in vitro* approach only mirrors partially the complex *in vivo* physiological conditions, our results support the idea of *ESAG* genotype selection as multifunctional tools for adaptation to the host, and VSG-ES remodelling as the molecular mechanism to achieve the expression of particular *ESAG* combinations. Further research about *ESAG* variability and function will be crucial to elucidate the role of these genes in parasite-host crosstalk. In addition, data on adaptation of other *T*. *b. gambiense* strains to mammalian sera would be useful to extend our observations to a more general context.

In the field, *T*. *b. gambiense* has been found in naturally infected livestock species such as goats and pigs [12,15]. Our results support the hypothesis that these animals are able to harbour *T*. *b. gambiense* for long-term periods. Molecular analyses revealed that pig serum ACLs displayed similar features to human serum ACLs, strengthening the hypothesis that swine are the main candidate to act as animal reservoirs of *T*. *b. gambiense* infection. However other animal species, either domestic or wild, should not be dismissed. 

These findings are relevant considering that HAT has been successfully controlled in several endemic foci and elimination is in progress in many areas [68]. Hence, a control strategy only based on detection and treatment of human cases would not guarantee the elimination of *T*. *b. gambiense* in endemic foci, where animal reservoirs could maintain the infection. 

## Materials and Methods

### Ethics statement

Human blood samples were taken from healthy donors, who provided written informed consent for the collection of samples. These samples were specifically obtained for this study. The procedure was approved by ethical committee of Institute of Parasitology and Biomedicine Lopez-Neyra (Spanish National Research Council). 

### In vitro culture of *Trypanosoma brucei gambiense*

Bloodstream forms of *Trypanosoma brucei gambiense* Eliane strain (MHOM/CI/52/ITMAP 2188) were used in all experiments [69]. This strain, originally isolated from an infected patient in Côte d’Ivoire (Ivory Coast) [39], was adapted to culture in HMI-9 medium [70], first supplemented with 1% normal human serum (NHS) and 20% foetal calf serum (FCS). After several months, NHS was no longer required and parasites effectively proliferated only with 20% FCS. To ensure the correct identification of our strain within *T*. *b. gambiense* group 1, we checked by PCR the presence of TgsGP [53] and LiTat 1.3 [71,72]. Cultures were maintained at 37 °C in humidified atmosphere containing 5% CO_2_. 

For adaptation assays, we maintained parasites in HMI-9 medium adding 20% of different mammalian sera (goat, pig, human and calf) with a starting density of 5 x 10^4^ parasites/ml. Goat and pig sera were obtained from Sigma-Aldrich^®^ whereas FCS was provided by Gibco^®^. We also tested NHS from Sigma-Aldrich^®^ but no viable cultures were obtained since parasites usually collapsed after 7-14 days. This drawback led us to use human serum from healthy donors. Different batches of NHS were used in order to avoid potential selection bias: the batch 1 was a pooled serum from several donors, the batch 2 and 3 came from two different individuals and the batch 4 was a mix of both 2 and 3 sera. Three independent adaptation experiments were performed for pig and goat sera. All sera were inactivated (56 °C for 30 minutes) before used. Cultures never reached densities over 2 x 10^6^ parasites/ml during the adaptation before being diluted. After 25 passages (75-105 days) in these media, we labelled the cultures as Adapted Cell Line (ACL) for the corresponding serum. We did not use clonal populations in this study since it was assumed that suspected variability inside population would speed up the selection of any involved gene. 

Disposable Neubauer chambers were used to manually count parasite density. We estimated duplication time for each ACL in their corresponding sera. The measure was carried out starting with 5 x 10^4^ parasites and counting at 48 hours in 24-well plaques in 1 ml of culture medium. 

To confirm the NHS resistance, we cloned by limiting dilution ACLs coming from pig, calf and goat sera. All clones tested (27 from calf, 17 from pig and 46 from goat sera) were fully resistant to 10% NHS, while sensitive *T*. *b. brucei* controls were systematically lysed within 16 hours, verifying the constitutive nature of the NHS resistance in *T*. *b. gambiense* [39]. This assay was conducted in 1 ml culture medium in 24-well plates with a starting density of 1 x 10^5^ parasites/ml. A cell line sensitive to NHS (*T*. *b. brucei* 1.2, clone 221a derived from Lister 427) was used as control to ensure the trypanolytic activity of the serum. 

### Antibodies anti-VSG production

The VSG expressed in the original line (LiTat 2.1) was purified by direct isolation from 4 x 10^8^ parasites following the protocol described elsewhere [73]. We obtained monoclonal antibodies (mAb) by inoculation of 50 ug of the isolated protein into BALB-C mice using standard immunization procedures (see for example 25). Six hybridomas were initially selected by standard monoclonal selection. The clone 2H5G3, which showed to be the most suitable for immunofluorescence (IF) and Western Blot (WB) analyses, was used for all assays. 

The VSG expressed in the first human serum ACL (named LiTat 3.1) obtained in our lab (HS1) was amplified from cDNA with primers annealing in splice leader sequence and in 3’ conserved region from all *T. brucei VSG*s [74]. RT-PCR product was cloned in pGEM^®^-T (Promega^©^) and sequenced. N-terminal domain was expressed as a fusion protein with histidine in Single Step (KRX) *E. coli* Competent Cells (Promega^©^) with a pET-28a vector (Novagen^®^) and induced with 0.1 mM IPTG and 0.1% rhamnose for 3 hours at 37°C. Soluble recombinant protein was purified with Ni Sepharose^TM^ 6 Fast Flow (GE Healthcare^©^) and inoculated into rabbits following standard immunization protocols. For both VSGs, peptide mass fingerprint was carried out to check their correspondence with nucleotide sequences. These sequences are available in GenBank database with KC257432 (LiTat 2.1) and KC257433 (LiTat 3.1) accession numbers. After several attempts we were unable to amplify *VSG*s corresponding to PS1, PS3 and GS3. As the primers used to amplify these genes were designed based on the *T*. *b. brucei* genome, slight sequence differences could hinder the sequencing of some *T*. *b. gambiense VSG*s. 

### Immunofluorescence

Cells were harvested by centrifugation at 1400 g for 10 minutes at room temperature, washed, and resuspended in TDB-glucose (KCl 5 mM, NaCl 80 mM, MgSO_4_ 1 mM, Na_2_HPO_4_ 20 mM, NaH_2_PO_4_ 2mM, glucose 20 mM, pH 7,4). 2 x 10^5^ cells were dried on slide, fixed in 1% paraformaldehyde (PFA) for 1 hour, washed with PBS, blocked with 0.5% blocking reagent (Roche^©^) and then incubated with mAb anti-LiTat 2.1 (1:10) and/or rabbit antiserum anti-LiTat 3.1 (1:5000) diluted in 0.5% blocking reagent. Alexa-Fluor^®^ 488 (anti-mouse) and Alexa-Fluor^®^ 594 (anti-rabbit) were used as secondary antibodies (Invitrogen^TM^). Cells were DAPI stained and visualized with a fluorescence microscope Zeiss, type Axio Imager A1, equipped with the AxioVision system. Images were mounted using ImageJ version 1.44 and Photoshop CS5 (Adobe Systems^©^) extended (version 12.1 x64).

### Genotyping

Total RNA was extracted from 5 x 10^7^ bloodstream form trypanosomes using High Pure RNA Isolation Kit (Roche^©^) according to the manufacturer’s recommendations. The amount of total RNA was quantified by spectrophotometric assay with NanoDrop^®^ system. cDNA was obtained from retrotranscription (RT) of 1 μg RNA using 300 nmol random primers in a 25 μl total volume with 100 units of SuperScript^TM^ III First-Strand Synthesis System (Invitrogen^TM^) and was conducted for 1 hour at 50°C. Partial open reading frames (ORF) from *ESAG3* and *ESAG6/7* genes were amplified from cDNA of each ACL (5U7:5 ’- TGTGCTGTTGGCTCTTTTGGGA-3’ and Tbg6/7L: 5’-CAGCACTCCCAACAATAAAACTGAAC-3’ for *ESAG6* and *ESAG7* including putative Tf binding sites and E3U: 5’-TCATGCAACACAAGGATGGT-3’ and E3L: 5’-TCCCCATATCCTTCGAATTA-3’ for *ESAG3*). 

Due to the lack of *T*. *b. gambiense* telomeres in databases, primers were designed from the published sequences of *T. brucei* TREU927. PCR was performed with 1 μl of cDNA, 1X buffer (10 mM Tris-HCl, 1.5 mM MgCl2, 50 mM KCl, pH 8.3), 100 μM of each dNTP, 0.5 μM of each primer, 1 U of Fast Start Taq DNA polymerase (Roche^©^) and double distilled water (DDW) until reaching 50 μl final volume. Samples were initially heated at 93 °C for 2 min and then submitted to 30 amplification cycles (93 °C for 30 seconds, 60 °C for 45 seconds and 72 °C for 90 seconds) followed by a final extension step at 72 °C for 5 minutes in a Bio-rad^©^ T100^TM^ Thermal Cycler. Genomic DNA of *T*. *b. gambiense* was amplified as positive control. RT negative controls were systematically integrated. RT-PCR products were cloned in pGEM^®^-T vector (Promega^©^) and sequenced. Sequences were manually visualized in order to check their integrity and ambiguous samples were removed from the analysis. Alignment was performed with Bioedit Sequence Alignment Editor 7.0.9.0. Sequences are available in GenBank with the following accession numbers: *ESAG7* genotypes [KC257410-KC257414], *ESAG6* genotypes: [KC257415-KC257418], *ESAG3* genotypes [KC257419-KC2574131]. Clustering trees were elaborated with sequenced samples using Neighbour-Joining method with MEGA4 software (Bootstrap=1000). Distances were computed using the Maximum Composite Likelihood method and they are in the units of the number of base substitutions per site. 

### Transferrin uptake

2 x 10^6^ trypanosomes were harvested and washed with TDB-glucose plus 1% BSA, resuspended in 250 µl in the same buffer and incubated for 10 minutes at 37 °C. Then, 5 µg of human holo-Tf Alexa Fluor^®^ 488-conjugated (Invitrogen^TM^) were added. Incubations were carried out for 0 (no Tf), 1, 2.5, 5 and 10 minutes at 37 °C. Cells were then fixed at least for one hour at 4 °C in 1% PFA diluted in cold PBS. Parasites were finally washed twice with PBS and labelled cells were analysed with a Becton Dickinson FACSCalibur^TM^ flow cytometer (BD Biosciences^©^) using BD CellQuest™ Pro version 4.0.2 software.

### Dextran uptake

Fluid-phase endocytosis was determined using Alexa Fluor® 488-labelled dextran 10,000 (Molecular Probes) as reported elsewhere [75]. 3 x 10^6^ parasites were resuspended in 10 μl of TDB-glucose plus 1% BSA and incubated at 37°C for 10 min. Alexa Fluor® 488-labelled dextran 10,000 was added at 2.5 mg/ml and cells were incubated 10 min at 37 °C. Then parasites were fixed at least for one hour at 4 °C in 1% PFA diluted in cold PBS. After washing twice with PBS, fluorescence was measured in flow cytometer.

### RT-qPCR

Quantitative PCR assays were performed in iCycler IQ^TM^ real-time PCR detection system (Bio-Rad Laboratories^©^); 20 µl reactions were set up containing 1 µl of cDNA (see Genotyping section), 0.5 μM of specific forward and reverse primers, 8 µl of DDW and 10 µl of PerfeCta^TM^ SYBR^®^ Green SuperMix for iQ (Quanta Biosciences^©^). Primers were designed in conserved regions of the *ESAG6/7* gene according to sequences obtained during genotyping analysis (qTbg6/7U: 5’-ATTTCCTTCGGTAGCTTGGG-3’ as common primer, qTbg6L: 5’-TTCACCACCCTCAACGTACA for *ESAG6* and qTbg7L: 5’-CACCGTATTCTCCCCCTTTT-3’ for *ESAG7*). For TbHpHbR (Tbg972.6.120) quantification, we designed the following primers: qTbHpHbRU (5’- AGCAGCTGCAGAGAAATGCT -3’) and qTbHpHbRL (5’- CTCTCACTGCTCCCACTGAA -3’).

The gene expression level was measured using the comparative cycle threshold CT method according to Pfaffl, 2001 [76]. The relative expression of different ACLs was normalized with *ATM* (PI3 Kinase-like -Tbb927.2.2260-) as housekeeping gene. Another housekeeping gene, *Myosin*, was included as control. All data were analyzed using Bio-Rad CFX Manager^TM^ software (version 1.6).

### Statistical analysis

We used Student´s t-test to assess significant differences in the growth of lines before and after adaptation and one-way ANOVA to compare the Tf uptake. Likelihood Ratio Test, completed with residues analysis, was used to detect significant serum-genotype associations. Confidence intervals (CI) were set at 99% in serum-genotypes associations and 95% in growth and Tf uptake experiments. IBM**^®^** SPSStatistics^®^ software (version 20) was used to perform all statistical analysis.

## Supporting Information

Figure S1 ***ESAG6/7* and *ESAG3* clustering trees.** Clustering tree of *ESAG6/7* (A) and *ESAG3* (B) genotypes found. The trees were constructed using the Neighbor-Joining method with partial ORF (673-676 bp of *ESAG6/7* and 853 bp of *ESAG3*). The percentage of replicate trees in which the associated taxa clustered together in the bootstrap test (1000 replicates) is shown next to the branches. Distances were computed using the Maximum Composite Likelihood method and are in the units of the number of base substitutions per site. *T*. *b. brucei* *ESAG*s 6, 7 and 3 are shown in red.
(TIF)

Figure S2 **Possible recombination scenarios in ACLs.** Schematic representation of different recombination events that might occur during adaptation process to mammalian sera tested. (A) Putative active VSG-ES of original cell line adapted to FCS, containing LiTat 2.1 *VSG* and probably without *ESAG*3 functional copies. In some ACLs as GS2, HS4 or GS3, recombination events could occur downstream the *ESAG*s 6 and 7 to another VSG-ES containing LiTat 3.1 *VSG* (B) or another unidentified *VSG* (C). Insertion of *ESAG*3 (and probably of other nearby *ESAG*s) could happen from inactive VSG-ESs or non-telomeric sites by gene conversion (D). This would explain the presence of the selected *ESAG*3 genotype found in HS3 in spite of the identical sequences and the same *VSG* found in this line respective to the original one. Alternatively, *in* *situ* switch might occur to a different VSG-ES, followed by a recombination downstream or upstream *ESAG*3 to a VSG-ES containing another *VSG* (E). This would entail a two steps scenario. However this possibility offers a suitable explanation for HS1, HS2 and all pig serum ACLs. Black arrows indicate the promoter sequence, the striped boxes are the 70-base-pair repeats upstream the *VSG* gene and white triangles represent telomeric repeats. Coloured boxes denote *ESAG* and *VSG* genes. The thick black line mirrors the transcribed genes. FCS: foetal calf serum, GS1/2/3: goat serum (adaptation experiments 1, 2 or 3), HS1/2/3/4: human serum (adaptation experiments 1, 2, 3 or 4), PS1/2/3: pig serum (adaptation experiments 1, 2 or 3). ACL: adapted cell line.
(TIF)

Table S1 **Summary of ESAG6/7, ESAG3 and VSG expressed by all ACLs.** Numbers represent the main genotype found in each ACL (see Tables 1 and 2 for further information). Frequency of the main genotype is given in parenthesis. If no number is shown, only the specified genotype was observed. Mixed: population contains more than 3 genotypes with no dominants. FCS: foetal calf serum, GS1/2/3: goat serum (adaptation experiments 1, 2 or 3), HS1/2/3/4: human serum (adaptation experiments 1, 2, 3 or 4), PS1/2/3: pig serum (adaptation experiments 1, 2 or 3). ACL: adapted cell line.
(DOC)
