# Supplementary figures and images for: Trypanosoma brucei gambiense Adaptation to Different Mammalian Sera Is Associated with VSG Expression Site Plasticity
Source: PLoS One. 2013 Dec 23;8(12):e85072. doi: 10.1371/journal.pone.0085072 (PMC3871602; doi:10.1371/journal.pone.0085072)

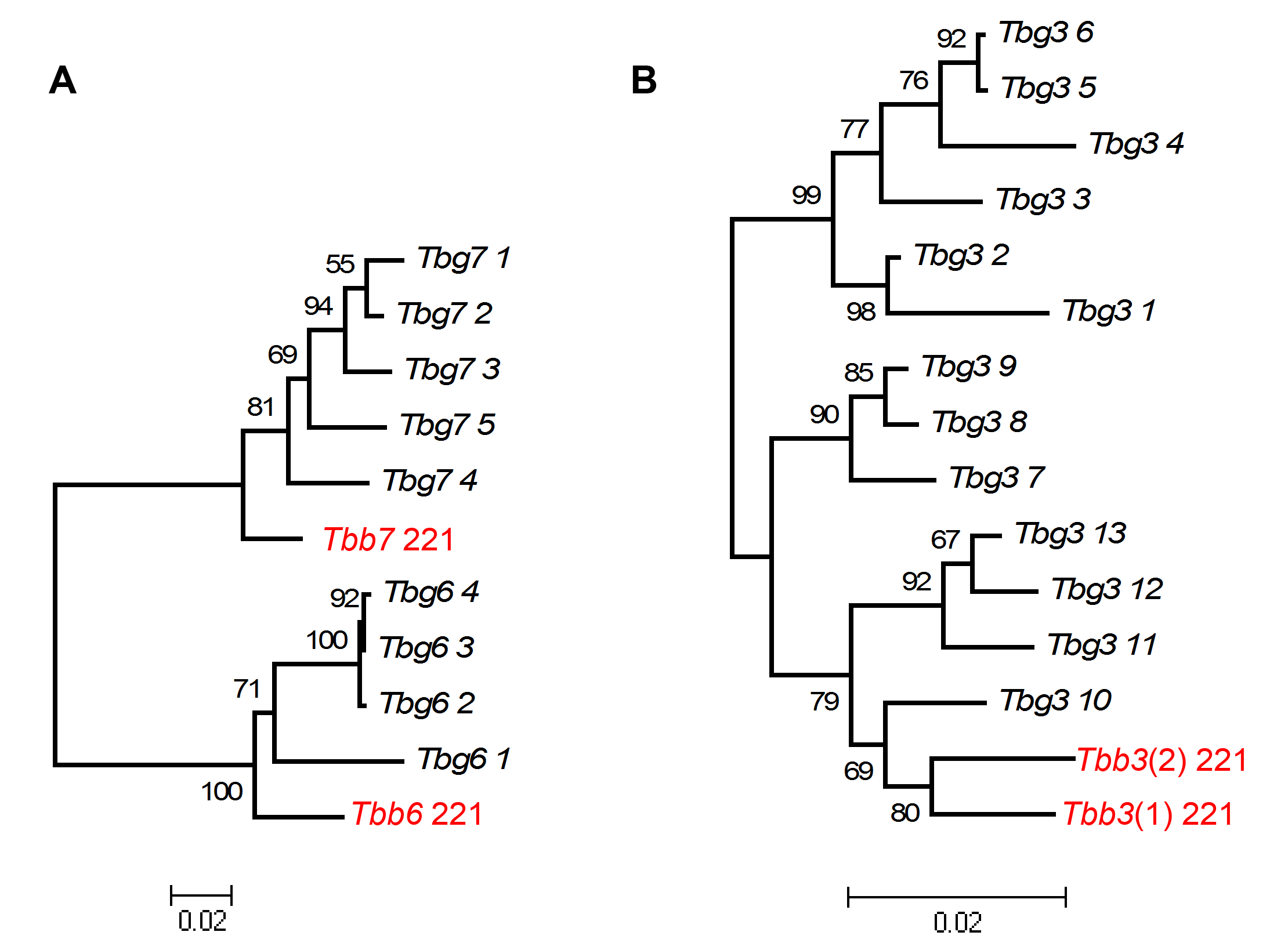

Supplement: Figure S1 — ESAG6/7 and ESAG3 clustering trees. Clustering tree of ESAG6/7 (A) and ESAG3 (B) genotypes found. The trees were constructed using the Neighbor-Joining method with partial ORF (673-676 bp of ESAG6/7 and 853 bp of ESAG3). The percentage of replicate trees in which the associated taxa clustered together in the bootstrap test (1000 replicates) is shown next to the branches. Distances were computed using the Maximum Composite Likelihood method and are in the units of the number of base substitutions per site. T. b. brucei ESAGs 6, 7 and 3 are shown in red. (TIF) [file pone.0085072.s001.tif]

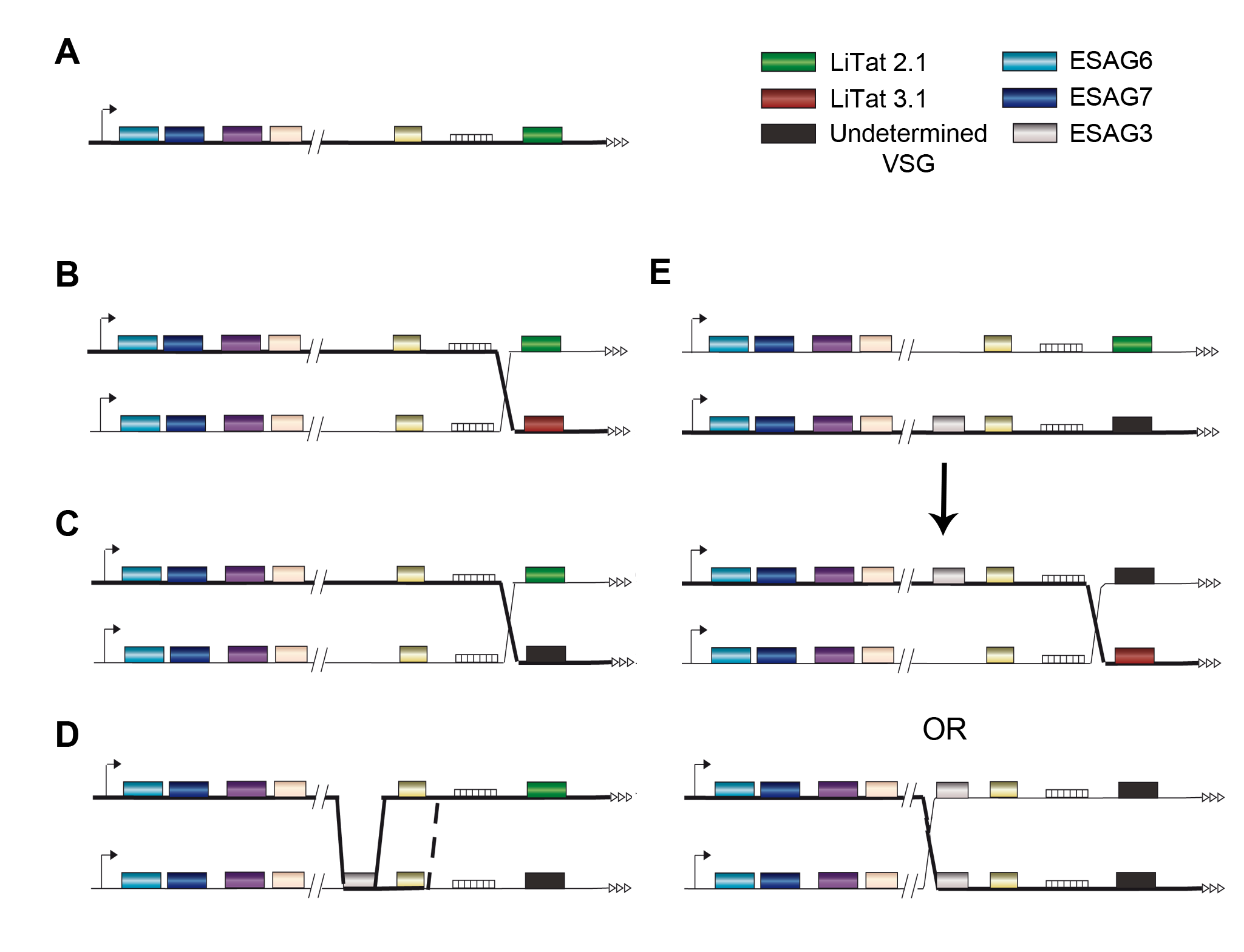

Supplement: Figure S2 — Possible recombination scenarios in ACLs. Schematic representation of different recombination events that might occur during adaptation process to mammalian sera tested. (A) Putative active VSG-ES of original cell line adapted to FCS, containing LiTat 2.1 VSG and probably without ESAG3 functional copies. In some ACLs as GS2, HS4 or GS3, recombination events could occur downstream the ESAGs 6 and 7 to another VSG-ES containing LiTat 3.1 VSG (B) or another unidentified VSG (C). Insertion of ESAG3 (and probably of other nearby ESAGs) could happen from inactive VSG-ESs or non-telomeric sites by gene conversion (D). This would explain the presence of the selected ESAG3 genotype found in HS3 in spite of the identical sequences and the same VSG found in this line respective to the original one. Alternatively, in situ switch might occur to a different VSG-ES, followed by a recombination downstream or upstream ESAG3 to a VSG-ES containing another VSG (E). This would entail a two steps scenario. However this possibility offers a suitable explanation for HS1, HS2 and all pig serum ACLs. Black arrows indicate the promoter sequence, the striped boxes are the 70-base-pair repeats upstream the VSG gene and white triangles represent telomeric repeats. Coloured boxes denote ESAG and VSG genes. The thick black line mirrors the transcribed genes. FCS: foetal calf serum, GS1/2/3: goat serum (adaptation experiments 1, 2 or 3), HS1/2/3/4: human serum (adaptation experiments 1, 2, 3 or 4), PS1/2/3: pig serum (adaptation experiments 1, 2 or 3). ACL: adapted cell line. (TIF) [file pone.0085072.s002.tif]
