# Supplementary material for: Trypanosoma brucei gambiense Adaptation to Different Mammalian Sera Is Associated with VSG Expression Site Plasticity
Source: PLoS One. 2013 Dec 23;8(12):e85072. doi: 10.1371/journal.pone.0085072 (PMC3871602; doi:10.1371/journal.pone.0085072)
Supplement: Table S1 — Summary of ESAG6/7, ESAG3 and VSG expressed by all ACLs. Numbers represent the main genotype found in each ACL (see Tables 1 and 2 for further information). Frequency of the main genotype is given in parenthesis. If no number is shown, only the specified genotype was observed. Mixed: population contains more than 3 genotypes with no dominants. FCS: foetal calf serum, GS1/2/3: goat serum (adaptation experiments 1, 2 or 3), HS1/2/3/4: human serum (adaptation experiments 1, 2, 3 or 4), PS1/2/3: pig serum (adaptation experiments 1, 2 or 3). ACL: adapted cell line. (DOC) [file pone.0085072.s003.doc]

Table S1. Summary of ESAG6/7, ESAG3 and VSG expressed by all ACLs.

| **ACL** | ***ESAG6* Genotype** | **ESAG6**  **Protein** | ***ESAG7***  **Genotype** | **ESAG7**  **Protein** | ***ESAG3***  **Genotype** | **ESAG3**  **Protein** | **VSG** |
| --- | --- | --- | --- | --- | --- | --- | --- |
| FCS | 3 (50) 4 (50) | 2/3/4 | 1 | 1/2 | Mixed | Mixed | 2.1 |
| GS1 | ND | ND | 3 (50) | 3 (50) | Mixed | Mixed | 3.1 |
| GS2 | 3 (60) | 2/3/4 | 1 (83.3) | 1/2 (83.3) | Mixed | Mixed | 3.1 |
| GS3 | 3 (50) | 2/3/4 (83.3) | 1 (62.5) | 1/2 (62.5) | Mixed | Mixed | ND_1 |
| HS1 | 2 (50)  3 (50) | 2/3/4 | 1 | 1/2 | 9 (75) | 9 (75) | 3.1 |
| HS2 | 4 | 2/3/4 | 1 (87.5) | 1/2 (87.5) | 9 | 9 | 3.1 |
| HS3 | 3 (50)  4 (50) | 2/3/4 | 1 | 1/2 | 9 (90.9) | 9 (90.9) | 2.1 |
| HS4 | 4 | 2/3/4 | 1 (91.7) | 1/2 (91.7) | Mixed | Mixed | 3.1 |
| PS1 | 4 | 2/3/4 | 5 | 5 | 1 | 1 | ND_2 |
| PS2 | 4 | 2/3/4 | 5 | 5 | 1 (92.8) | 1 (92.8) | 3.1 |
| PS3 | ND | ND | 5 (93.3) | 5 (93.3) | 13 | 13 | ND_3 |

**﻿**
